# Plasticity of BRCA2 Function in Homologous Recombination: Genetic Interactions of the PALB2 and DNA Binding Domains

**DOI:** 10.1371/journal.pgen.1002409

**Published:** 2011-12-15

**Authors:** Nicolas Siaud, Maria A. Barbera, Akinori Egashira, Isabel Lam, Nicole Christ, Katharina Schlacher, Bing Xia, Maria Jasin

**Affiliations:** 1 Developmental Biology Program, Memorial Sloan-Kettering Cancer Center, New York, New York, United States of America; 2 Louis V. Gerstner Sloan-Kettering Graduate School of Biomedical Sciences, Memorial Sloan-Kettering Cancer Center, New York, New York, United States of America; 3 Department of Radiation Oncology, The Cancer Institute of New Jersey, Robert Wood Johnson Medical School, University of Medicine and Dentistry of New Jersey, New Brunswick, New Jersey, United States of America; University of Washington, United States of America

## Abstract

The breast cancer suppressor BRCA2 is essential for the maintenance of genomic integrity in mammalian cells through its role in DNA repair by homologous recombination (HR). Human BRCA2 is 3,418 amino acids and is comprised of multiple domains that interact with the RAD51 recombinase and other proteins as well as with DNA. To gain insight into the cellular function of BRCA2 in HR, we created fusions consisting of various BRCA2 domains and also introduced mutations into these domains to disrupt specific protein and DNA interactions. We find that a BRCA2 fusion peptide deleted for the DNA binding domain and active in HR is completely dependent on interaction with the PALB2 tumor suppressor for activity. Conversely, a BRCA2 fusion peptide deleted for the PALB2 binding domain is dependent on an intact DNA binding domain, providing a role for this conserved domain in vivo; mutagenesis suggests that both single-stranded and double-stranded DNA binding activities in the DNA binding domain are required for its activity. Given that PALB2 itself binds DNA, these results suggest alternative mechanisms to deliver RAD51 to DNA. In addition, the BRCA2 C terminus contains both RAD51-dependent and -independent activities which are essential to HR in some contexts. Finally, binding the small peptide DSS1 is essential for activity when its binding domain is present, but not when it is absent. Our results reveal functional redundancy within the BRCA2 protein and emphasize the plasticity of this large protein built for optimal HR function in mammalian cells. The occurrence of disease-causing mutations throughout BRCA2 suggests sub-optimal HR from a variety of domain modulations.

## Author Summary

The breast tumor suppressor BRCA2 has a major role in DNA repair by homologous recombination (HR). BRCA2 is a large protein with multiple domains that interact with several proteins as well as with DNA, complicating our understanding of how the protein functions in cells. To investigate the mechanism by which BRCA2 functions in HR in cells, we created fusions consisting of various BRCA2 domains and also introduced mutations into these domains to disrupt specific protein and DNA interactions. We find that DNA binding by BRCA2 is critical when a BRCA2 peptide is deficient in binding another breast cancer suppressor, PALB2, but not when the peptide can bind PALB2, suggesting alternative mechanisms of activity. Binding the small peptide DSS1 is also essential for HR only in some contexts, as are activities in the BRCA2 C terminus. Our results reveal redundancy of BRCA2 domains and emphasize plasticity within this large protein built for optimal HR function in mammalian cells. The occurrence of disease-causing mutations throughout BRCA2 suggests sub-optimal HR from a variety of domain modulations.

## Introduction

Precise repair of DNA double-strand breaks (DSBs) in cells is dependent on homologous recombination (HR), a mechanism that allows the use of an intact, identical DNA molecule (usually the sister chromatid) to restore the correct sequence at the site of the DSB [1], [2]. Central to HR reactions is DNA strand exchange catalyzed by the RAD51 protein. RAD51 forms a nucleoprotein filament with single-strand DNA (ssDNA) generated by DNA end resection [3], which then invades a homologous duplex DNA to initiate HR.

In the cellular context, RAD51 requires several cofactors for its activity [1]. Proteins termed mediators are critical for RAD51 to overcome the inhibition to filament formation posed by the high affinity ssDNA binding protein RPA which otherwise obstructs RAD51 access to DNA. In mammalian cells, the breast and ovarian tumor suppressor BRCA2 is postulated to be a key mediator protein. BRCA2-deficient rodent and human cells are deficient in HR [4] and have defects in forming DNA damage-induced RAD51 nuclear foci [5]. Recent studies have illuminated the biochemical properties of BRCA2 [6]–[8]. BRCA2 promotes RAD51 filament assembly specifically onto ssDNA, rather than dsDNA, allowing it to displace RPA. Further, BRCA2 stabilizes RAD51 filaments by preventing ATP hydrolysis.

**Figure 1:**
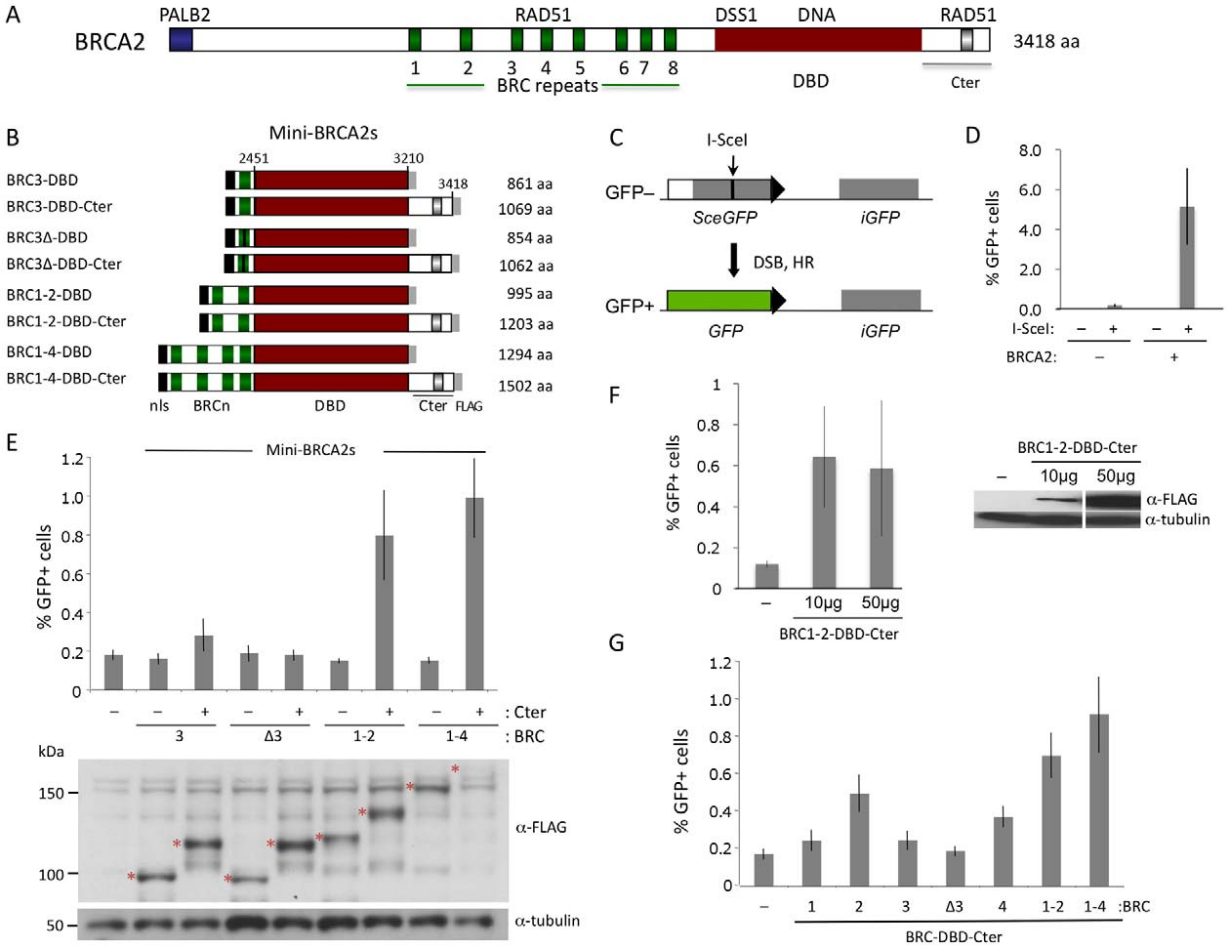
HR proficiency of mini-BRCA2 peptides. A. Human BRCA2 domain structure. BRCA2 binds RAD51 at the eight BRC repeats in the central region of the protein and at a distinct site in the C-terminus (Cter). In addition, BRCA2 binds PALB2 at the N terminus and has a conserved DSS1 and DNA-binding domain (DBD). B. Mini-BRCA2 domain structures. Peptides contain the indicated BRCA2 domains as well as a nuclear localization signal (nls) at the N terminus and a FLAG epitope tag at the C terminus. C. DR-GFP. The DR-GFP reporter consists of two defective *GFP* genes [29]. Expression of I-SceI endonuclease results in a double-strand break (DSB) at the I-SceI site in the *SceGFP* gene which can be repaired using the homologous sequence in the *iGFP* gene to generate GFP positive cells which are quantified by flow cytometry. D. BRCA2-complemented V-C8 cells have substantially higher DSB-induced HR than uncomplemented cells. *P* = 0.0002, two-tailed unpaired t test. A bacterial artificial chromosome (BAC) (clone RP11-777I19; BACPAC Resource Center at the Children's Hospital Oakland Research Institute in Oakland, California) which contains human *BRCA2* [53] was stably introduced into V-C8 DR-GFP cells [12] using a linked neomycin resistance gene for selection. BRCA2 expression was verified by Western blot analysis (not shown). E. Mini-BRCA2s containing the Cter partially correct the HR defect of *Brca2*-deficient V-C8 hamster cells. Mini-BRCA2s that have activity relative to no mini-BRCA2 are BRC3-DBD-Cter (*P*<0.05), and BRC1–2-DBD-Cter and BRC1–4-DBD-Cter (*P*≤0.001; unpaired *t* test). All peptides (red asterisks) are of the expected sizes. BRC1–4-DBD-Cter corrects the HR defect as well or better than BRC1–2-DBD-Cter despite being poorly detected. F. Comparison of HR activity at different levels of BRCA2 peptide expression. The BRC1–2-DBD-Cter expression vector was transfected at the usual amount (50 µg) or at a reduced amount (10 µg). HR levels were similar in both cases (*P* = 0.82). G. Mini-BRCA2s with multiple BRC repeats are more active in HR than those with single BRC repeats. Relative to BRC1–2-DBD-Cter, *P*≤0.0001 for single repeat mini-BRCA2s, except for BRC2-DBD-Cter where *P* = 0.003. All mini-BRCA2s have significant activity relative to no mini-BRCA2 (*P*≤0.005), except BRCΔ3-DBD-Cter (*P* = 0.248).

Human BRCA2 is 3418 amino acids and consists of multiple domains whose relative contributions to HR are somewhat ambiguous [2] (Figure 1A). The central portion of the protein contains eight diverged repeats of ∼35 amino acids, termed “BRC”, which bind RAD51 monomers [9]. When expressed in cells either individually or as sets of repeats, the BRC repeats interfere with HR [10]–[12], presumably by acting as a mimic of RAD51 self association [13]. In the context of the full-length protein, however, the BRC repeats promote RAD51 filament assembly on ssDNA [6]–[8]. An additional RAD51 interacting site is found within the ∼200 amino acid C terminus (Cter) of BRCA2 encoded by exon 27 [14]. Unlike the BRC repeats, the Cter does not bind RAD51 monomers; instead, it interacts with two adjacent RAD51 protomers in filaments on ssDNA to stabilize them [15], [16]. Isolated from the intact protein, a Cter peptide interferes with HR *in vivo* [17], although in the context of otherwise intact BRCA2, the Cter region promotes HR [4]. RAD51 binding to a site within the Cter is regulated by CDK phosphorylation of residue S3291 at G2/M phase [17], which destabilizes RAD51 filaments, and is crucial to at least two processes that are independent of HR-mediated repair, i.e., maintaining the integrity of nascent strands at stalled replication forks [18] and preventing premature entry into mitosis [19].

A large segment of BRCA2 encompasses a DNA binding domain (DBD) [20]. The DBD contains oligonucleotide/saccharide (OB) folds found in ssDNA binding proteins like RPA, as well as a long helical domain with a putative dsDNA binding domain at its apex. Facing away from DNA, the 70 amino acid protein DSS1 also binds to the BRCA2 DBD. DSS1 promotes RAD51 focus formation upon DNA damage in mammalian cells [21] and also promotes HR in organisms that do not have a BRCA2 ortholog [22]. At its N terminus, BRCA2 binds the PALB2 protein, which also promotes HR [23]. PALB2, like BRCA2, is a breast cancer suppressor and is also associated with Fanconi anemia, a developmental and tumor predisposition syndrome [2], [24]–[26].

Because significant portions of the identified activities of BRCA2 are dedicated to binding RAD51 and ssDNA, we previously constructed fusion proteins in which one or more BRC repeats were fused directly to a subunit of RPA [12]. Such fusion peptides, consisting of as little as 2% of BRCA2, are functional in promoting HR, implying a primary function for BRCA2 is to deliver RAD51 to ssDNA.

Given the complex domain structure of BRCA2 and the diverse domain compositions of BRCA2 orthologs in other organisms [27], we sought to comprehensively investigate the role of BRCA2 domains in HR. We find that mutations that disrupt DNA binding in the DBD significantly reduce or abolish HR when PALB2 binding is absent, while deletion of the entire DBD has little impact on HR when PALB2 binding is present. Moreover, mutation of a DSS1-binding residue in the DBD abolishes HR when the DBD is present, even though deletion of the DBD is well tolerated. We found that the BRCA2 Cter contributes to HR through both RAD51-dependent and independent activities, suggesting additional functions for this domain to those identified thus far. Lastly, a “micro-BRCA2” peptide <20% the size of full-length BRCA2 is functional in HR. Thus, our results highlight a previously unappreciated plasticity within the BRCA2 protein to promote optimal HR repair in cells.

## Results

### Mini-BRCA2 peptides proficient in HR *in vivo*

To determine whether subdomains of BRCA2 could be identified which are active in HR *in vivo*, we first generated a series of peptides consisting of BRC repeats (BRC3, BRC1–2, and BRC1–4) fused with the BRCA2 DBD [20] (Figure 1B). This same set of BRC repeats was previously fused to RPA70 to generate peptides active in HR [12]. Because the Cter domain located downstream of the BRCA2 DBD has proved to be important for HR [4], we created a parallel set of fusion proteins containing the Cter. Collectively, we termed these peptide fusions “mini-BRCA2s.”

HR activity of the mini-BRCA2s was tested in a *Brca2* mutant V-C8 hamster cell line [28] containing an integrated copy of the DR-GFP reporter [12]. This reporter measures HR repair of an I-SceI endonuclease-generated DSB in the chromosome by the restoration of an intact green fluorescent protein (GFP) gene [29] (Figure 1C). V-C8 cells have a ∼20-fold reduction in HR compared with either wild-type cells [12] or V-C8 cells complemented with full-length BRCA2 (Figure 1D). Mini-BRCA2s were transiently expressed together with I-SceI endonuclease in V-C8 cells and found to be active in supporting HR (Figure 1E). However, activity in the *Brca2* mutant cells requires the presence of the Cter. For example, BRC1–4-DBD-Cter increases HR ∼5-fold over background levels, whereas BRC1–4-DBD is completely inactive. Similar results were obtained for BRC1–2-DBD-Cter and BRC1–2-DBD, respectively. Although the peptides can be expressed at somewhat different levels, expression level does not noticeably affect HR activity (Figure 1F). These results indicate that the BRCA2 DBD is not equivalent to RPA in promoting HR. Further, these results demonstrate the importance of the Cter in HR *in vivo*, contrasting with results obtained *in vitro* where the Cter is not required for recombination activity [30].

In addition to the Cter, we find that mini-BRCA2s differ from BRC-RPAs in another important aspect: Mini-BRCA2s are less active in HR if they contain only a single BRC repeat. Thus, BRC3-DBD-Cter is marginally active, increasing HR levels only 1.5-fold above background (Figure 1E), while BRC3-RPA activity is substantial [12]. The small increase in HR nevertheless depends on the ability of the BRC repeat to bind RAD51, as BRC3Δ-DBD-Cter, which contains a deletion of conserved BRC residues [9], [11], is completely inactive (Figure 1E). As the eight BRC repeats share only limited conservation and bind RAD51 with different affinities [31] and properties [32], we further tested other single BRC repeat mini-BRCA2s. We found that both BRC2-DBD-Cter and BRC4-DBD-Cter are more active than BRC1-DBD-Cter and BRC3-DBD-Cter, but that neither is as active as the multiple BRC repeat mini-BRCA2s (Figure 1G). These results indicate that higher affinity or valency of RAD51 binding is more critical for mini-BRCA2 function than for BRC-RPA function, further distinguishing mini-BRCA2 and BRC-RPA function.

### Mini-BRCA2 function in HR requires an intact DBD

Mini-BRCA2s active in HR are significantly smaller than full length BRCA2 – about one third the size – yet they are still comprised of a number of domains with multiple biochemical activities and interacting partners. To determine whether mini-BRCA2s could be further simplified, individual domain requirements were tested. Because BRC1–2-DBD-Cter is nearly as efficient as BRC1–4-DBD-Cter in HR but is more easily detected on Western blots, it was used to assess the importance of these other domains. For simplicity, we refer to BRC1–2-DBD-Cter below as “mini-BRCA2”.

**Figure 2:**
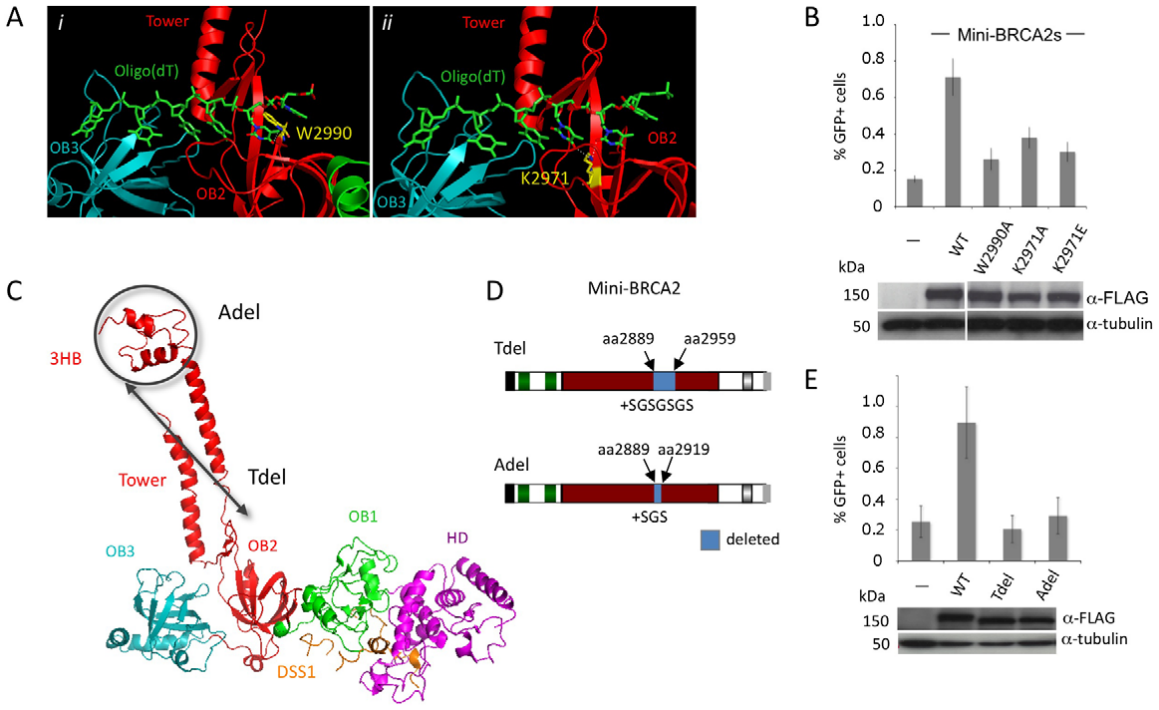
Mini-BRCA2 activity requires an intact DBD. A. BRCA2-ssDNA interface in the DBD. BRCA2 OB2 (red) and OB3 (teal) folds interact with oligo(dT) (green) [20]. *i)* A key interaction in OB2 is at a tryptophan residue (gold) (W2990 human/W2909 mouse) which stacks with the first two oligo(dT) bases, in parallel with the first base and at an angle with the second. *ii)* A lysine (gold) (K2971 human/K2891 mouse), also in OB2, interacts with the edges of the second and third bases. For the relevant interacting nucleotides and lysine, the nitrogens and oxygens are blue and red, respectively. Dotted white lines indicate the interaction. Detail of the mouse BRCA2 DBD^ΔTower^-DSS1-oligo(dT) ternary complex [20] extracted from the RCSB Protein Data Bank, accession code 1MJE. B. Mutations of ssDNA binding residues in mini-BRCA2 impair HR. The mini-BRCA2 in these assays is BRC1–2-DBD-Cter. For values relative to wild-type mini-BRCA2, *P*≤0.0001; for values relative to no mini-BRCA2, *P*≤0.002; for W2990A compared with K2971A, *P* = 0.0061. Western blots of the transiently expressed mini-BRCA2s are shown beneath the graph. C. BRCA2 DBD crystal structure showing the tower domain arising from OB2, with a three-helix bundle (3HB) at the apex of the tower which is proposed to bind dsDNA [20]. Deletions which remove a large part of the tower (Tdel) or only the 3HB (Adel) are indicated on the structure. The structure of the DBD was solved in a co-crystal with the 70 amino acid protein DSS1 (orange). DSS1 binds in an extended conformation to the helical domain (HD, magenta) and OB1 (green), at the opposite side of the structure from oligo(dT) (not shown). Mouse BRCA2 DBD-DSS1 structure extracted from the RCSB Protein Data Bank, accession code 1MIU. D. Domain structures of mini-BRCA2s modified within the tower. For Tdel, the seven amino acid linker provides flexibility for the first tower helix and the disordered region to fold back and reconnect with OB2. For Adel, the three amino acid linker at the site of the deletion is predicted to connect the ends of the tower without distorting the structure. Boundaries of BRCA2 amino acids deleted are indicated. E. Deletion of the tower domain or just the 3HB in mini-BRCA2 abrogates HR. For values relative to wild-type mini-BRCA2, *P*≤0.001; relative to no mini-BRCA2, neither Tdel nor Adel is statistically significant (*P* = 0.53 and 0.58, respectively).

The BRCA2 DBD is suggested to be critical for function in HR through its ssDNA binding activity [20]. The DBD contains three OB folds [20], which are frequent in ssDNA binding proteins [33]. A co-crystal of the BRCA2 DBD with oligo(dT) demonstrates binding primarily to OB2, including through interactions similar to RPA bound to ssDNA [20]. For example, a tryptophan (W2990) in BRCA2 OB2 is sandwiched between two bases (Figure 2A*i*), similar to a phenylalanine at the same position in RPA, and a lysine (K2971) interacts with adjacent bases in the structure through hydrogen bonding (Figure 2A*ii*). We mutated these residues in mini-BRCA2 and tested the mutants for HR proficiency in the V-C8 cells. Mutation of either residue, especially W2990, significantly reduces HR compared with wild-type mini-BRCA2 (Figure 2B), indicating that ssDNA binding is critical for mini-BRCA2 function. HR is not completely abolished with either single mutant, consistent with multiple contacts between the DBD and ssDNA. These results demonstrate the importance of ssDNA-contact residues for BRCA2 DBD function.

In addition to OB folds, the BRCA2 DBD has a three-helix bundle (3HB) located at the apex of an unusual “tower” domain extending from OB2 (Figure 2C). The 3HB is similar to dsDNA-binding folds and has been implicated in dsDNA binding *in vitro* [20]. It has been proposed that nucleation of RAD51 filaments occurs through BRCA2 bound to ssDNA:dsDNA junctions, which would explain a role for dsDNA binding in HR reactions [34]. To test the requirement for the putative dsDNA binding domain, a mini-BRCA2 was constructed that is deleted for a portion of the tower (Tdel; Figure 2C, 2D). The Tdel deletion is based on the same BRCA2 tower deletion previously used for crystallization [20]; a flexible peptide linker was added to span the distance found in the crystal structure between the deleted amino acids to attempt to maintain the overall structural integrity of the DBD. We found that Tdel is unable to correct the HR defect of the V-C8 cells (Figure 2E). To more precisely explore the requirement for the BRCA2 tower domain in HR, the Adel mutation was created in which only the 3HB at the apex of the tower was deleted (Figure 2D). A flexible peptide linker was added when constructing Adel to span the distance between the deleted amino acids. As with Tdel, Adel is not able to correct the HR defect of V-C8 cells (Figure 2E). Although we cannot formally rule out the possibility that the deletions perturb the integrity of the OB2 domain, these results support a role for the BRCA2 3HB in HR, possibly through interaction with dsDNA.

### Mini-BRCA2 function in HR requires intact DSS1 and RAD51 Cter binding sites

**Figure 3:**
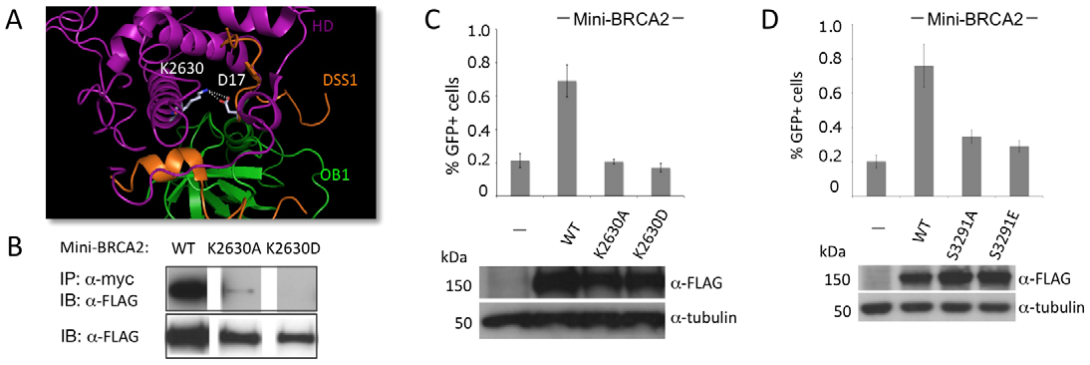
Mutation of a DSS1-interacting residue or the Cter RAD51-interacting residue S3291 significantly reduces the HR activity of mini-BRCA2. A. Detail of the BRCA2-DSS1 interface. BRCA2 and DSS1 interact through both charged and hydrophobic residues [20]. For the former, basic residues in BRCA2 interact with acidic residues in DSS1 (e.g., K2630 human/K2551 mouse interacts with DSS1 D17; white). Detail of the mouse BRCA2 DBD-DSS1 structure [20] extracted from the RCSB Protein Data Bank, accession code 1MIU. B. Mutation of BRCA2 K2630 in mini-BRCA2 reduces DSS1 interaction. Immunoprecipitation of myc-tagged DSS1 brings down FLAG-tagged wild-type mini-BRCA2 but K2630A or K2630D-mutated mini-BRCA2 to a much lower or undetectable level, respectively. C. Mutation of a DSS1 interacting residue in mini-BRCA2 abrogates HR. For values relative to wild-type mini-BRCA2, *P*≤0.001; relative to no mini-BRCA2, neither K2630A nor K2630D is statistically significant. D. Mutation of the Cter RAD51 binding site in mini-BRCA2 impairs HR. For values relative to wild-type mini-BRCA2, *P*≤0.0001; relative to no mini-BRCA2, *P*≤0.0001.

The BRCA2 DBD binds several proteins, including the small peptide DSS1 [35], which is reported to promote RAD51 focus formation after DNA damage [21]. In the co-crystal structure, DSS1 interacts with the DBD at several residues, in part through a series of charge interactions [20]. We chose to disrupt one of the charge interactions, mutating residue K2630 which interacts with DSS1 residue D17 (Figure 3A). Mutation of this DSS1-interacting residue in mini-BRCA2 to a neutral amino acid (K2630A) or to an amino acid of opposing charge (K2630D) results in markedly reduced or undetectable co-immunoprecipitation with DSS1, respectively (Figure 3B). Further, both mutations abolish HR proficiency *in vivo* (Figure 3C), providing evidence that interaction with DSS1 is important for mini-BRCA2 function.

The BRCA2 Cter binds RAD51 at a site which is not homologous to the BRC repeats and which appears to bind RAD51 differently, having a preference for RAD51 nucleoprotein filaments as opposed to RAD51 monomers [15], [16]. Moreover, this site is subject to regulated phosphorylation which blocks interaction with RAD51 [17]. We created two mutations at the interaction site which abrogate RAD51 binding, S3291A and the phosphorylation mimic S3291E [17], and found that both substantially reduce the HR activity of the mini-BRCA2 (Figure 3D). These results suggest that two modes of RAD51 binding – at the BRC repeats and at the Cter – are important for mini-BRCA2 activity in HR.

In summary, this series of mutations within the various domains of mini-BRCA2 provides evidence that binding to ssDNA, dsDNA, DSS1, and RAD51 (in two modes) are all required for mini-BRCA2 function in HR *in vivo*.

### PALB2-binding increases the HR proficiency of BRCA2 fusion peptides

**Figure 4:**
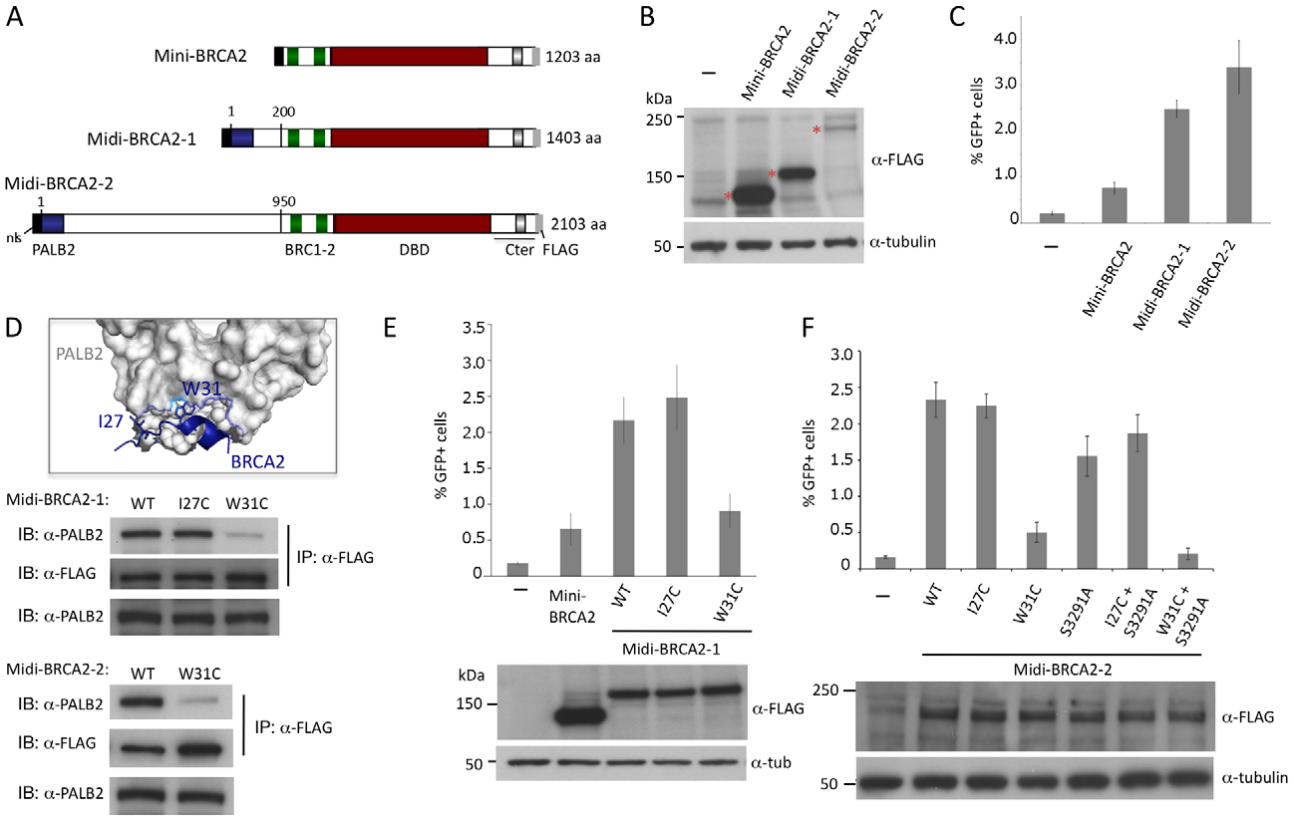
Midi-BRCA2s containing an intact PALB2 binding site restore HR levels. A. Midi-BRCA2 domain structures. Midi-BRCA2s contain the PALB2-interacting domain at the N terminus. Midi-BRCA2-2 differs from full-length BRCA2 primarily in being deleted for the last 6 BRC repeats; it also contains a small deletion N-terminal to BRC1. B. Western blot analysis showing expression of the transiently expressed mini and midi-BRCA2s. C. Midi-BRCA2s are more proficient at HR than mini-BRCA2. Relative to mini-BRCA2, *P*≤0.0001 for midi-BRCA2s. D. BRCA2 W31 is a key PALB2 binding residue. Mutation of midi-BRCA2 W31 disrupts binding to PALB2. Immunoprecipitation of FLAG-tagged wild-type or I27C midi-BRCA2 efficiently brings down PALB2 while FLAG-tagged W31C midi-BRCA2 precipitation of PALB2 is significantly impaired. BRCA2 W31 extends from a short helix into a hydrophobic pocket of the PALB2 ß-propeller; the indole nitrogen (light blue) also supports a polar interaction with a water bridge to PALB2 S1065 in the PALB2 pocket (dotted line) [36] (accession code 3EU7). Only a portion of the PALB2 ß-propeller is shown. E. Mutation of the PALB2 binding site (W31C) in midi-BRCA2-1 reduces but does not abolish HR activity. For values relative to wild-type midi-BRCA2-1, *P*≤0.0001 except for midi-BRCA2 I27C, which is not significant; for mini-BRCA2 relative to midi-BRCA2-1 W31C, *P* = 0.13. Western blot analysis shows expression of the transiently expressed mini and midi-BRCA2s. F. Mutation of the PALB2 binding site (W31C) in midi-BRCA2-2 reduces but does not abolish HR activity. Relative to wild-type midi-BRCA2-2, *P*≤0.005 for the W31C and/or S2391A mutants; for midi-BRCA2-2 W31C relative to no midi-BRCA2-2, *P*≤0.0001 whereas W31C+S3291A is not significantly different from no midi-BRCA2-2 (*P* = 0.13). Western blot analysis shows expression of the transiently expressed peptides.

Mini-BRCA2s are functional for HR but they only partially restore HR levels to BRCA2-deficient V-C8 cells. Mini-BRCA2s are missing the N-terminal ∼30% of BRCA2, including the interaction site for the PALB2 protein found in the first ∼50 amino acids of BRCA2 [23]. Given that mutations that impair PALB2 binding impair the ability of full-length BRCA2 to function in HR [23], we added the PALB2 interaction site to mini-BRCA2, forming what we termed midi-BRCA2-1 (Figure 4A). We also added a longer segment so that the N-terminal region of BRCA2 was largely restored (midi-BRCA2-2). Midi-BRCA2s are expressed at the expected sizes, although the level of midi-BRCA2-2 detected on Western blots is low compared with either midi-BRCA2-1 or mini-BRCA2 (Figure 4B). Both midi-BRCA2-1 and midi-BRCA2-2 complement the HR defect of V-C8 cells to a greater extent than mini-BRCA2, leading to a 3 to 4-fold higher level of HR (Figure 4C) and approaching that of full-length BRCA2 (Figure 1D).

To determine if the higher level of HR is due to interaction with PALB2, we disrupted the PALB2 binding site of the midi-BRCA2s. BRCA2 W31 is a key contact residue with PALB2, projecting into a hydrophobic pocket of the PALB2 ß-propeller (Figure 4D, [36]). W31C mutation, which is annotated in the Breast Cancer Information Core (BIC) database, impairs the interaction of full-length BRCA2 with PALB2 and impairs HR function [23]. By contrast, mutation of BRCA2 residue I27 does not impact binding to PALB2 or HR. Similarly, we find that W31C mutation of either midi-BRCA2-1 or midi-BRCA2-2 reduces binding to PALB2 whereas I27C mutation does not (Figure 4D; data not shown), while neither mutation affects the expression of the midi-BRCA2s (Figure 4E, 4F). Importantly, W31C mutation markedly reduces the ability of the midi-BRCA2s to function in HR, whereas I27C mutation does not (Figure 4E, 4F). These results indicate that interaction of the midi-BRCA2s with PALB2 promotes HR. HR is not completely abolished by W31C mutation, however, as HR activity for either of the midi-BRCA2 mutants is similar to that of mini-BRCA2 (Figure 4E, 4F). Combined mutation of the Cter RAD51 binding site (S3291A) and W31C, however, severely compromises midi-BRCA2-2 function in HR (Figure 4F), indicating that when the PALB2 binding site is disrupted, the residual activity in the midi-BRCA2-2 requires the Cter RAD51 binding site for activity, as does mini-BRCA2 (Figure 2I).

### Interaction with PALB2 partially compensates for mutations in domains essential for mini-BRCA2 HR activity

**Figure 5:**
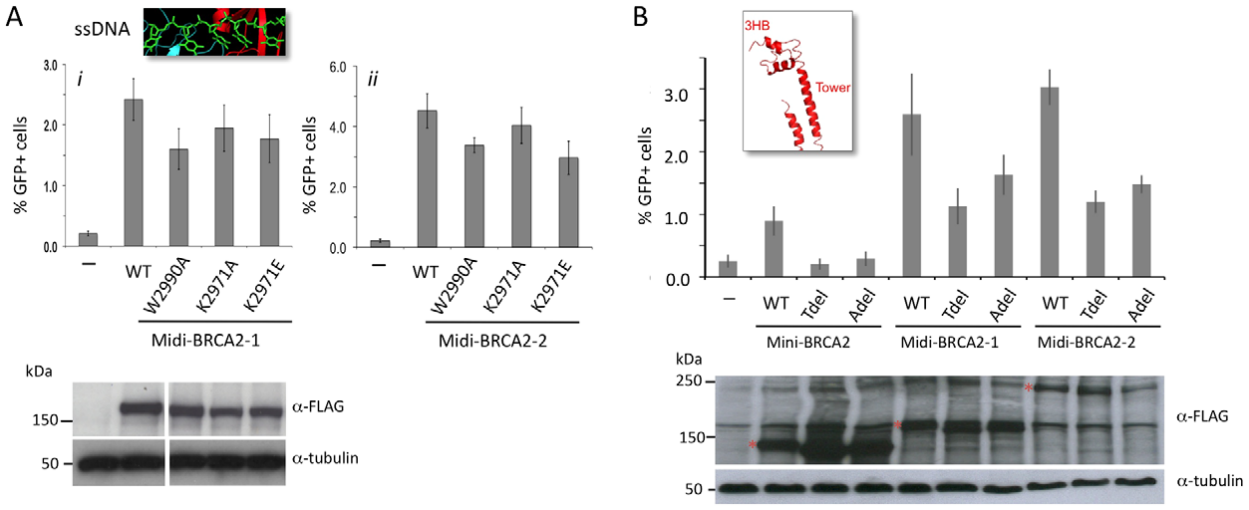
Interaction with PALB2 partially compensates for mutations in the DNA binding domain. A. Midi-BRCA2s with mutations in ssDNA contact residues retain substantial HR activity. *P*≤0.0001 compared with no BRCA2 peptide. *i)* Compared with wild-type midi-BRCA2-1, *P* values are 0.002 (W2990A), 0.05 (K2971A), and 0.01 (K2971E). *ii)* Compared with wild-type midi-BRCA2-2, *P* values are 0.0004 (W2990A), 0.15 (K2971A), and 0.0002 (K2971E). Western blot analysis shows expression of the transiently expressed peptides. B. Midi-BRCA2s with deletion of the tower domain or just the three-helix bundle retain partial HR activity. *P*≤0.0002 compared with no BRCA2 peptide. Compared with the respective wild-type proteins, *P* values for mini-BRCA2 mutations are 0.003 (Tdel) and 0.0008 (Adel) (Figure 2E), for midi-BRCA2-1 are 0.002 (Tdel) and 0.02 (Adel), and for midi-BRCA2-2 are 0.0001 (Tdel, Adel). Western blot analysis shows expression of the transiently expressed peptides.

Given that the PALB2 binding site has a strong effect on HR activity, we asked how this effect is modulated by activities from the DBD. In the context of PALB2 binding, substitution mutations in ssDNA binding residues in DBD OB2 in midi-BRCA2s decrease HR <50% (Figure 5A), whereas these same mutations in mini-BRCA2 reduce HR close to background levels (note especially W2990A; Figure 2B). Likewise, the Tdel and Adel mutations in the DBD tower domain modulating dsDNA interaction only mildly affect HR activity of midi-BRCA2s (∼50% HR reduction, Figure 5B) but completely abolish HR activity of mini-BRCA2 (Figure 2E). These results indicate that the ability of the midi-BRCA2s to interact with PALB2 partially compensates for disruption of ssDNA and dsDNA binding, both of which otherwise become essential for HR activity.

**Figure 6:**
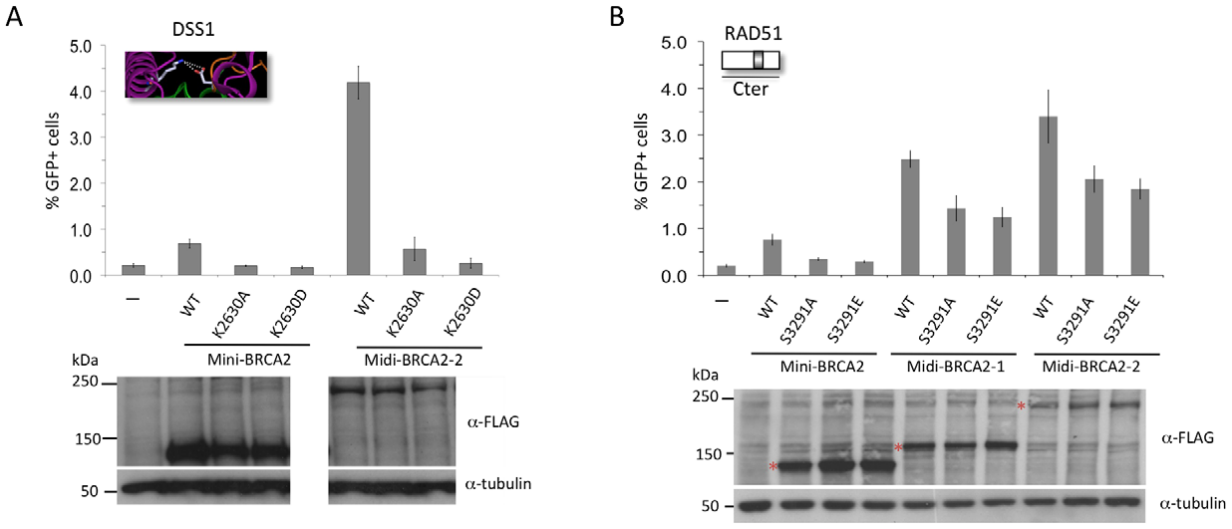
Interaction with PALB2 partially compensates for point mutations in the Cter that interfere with RAD51 binding, but cannot compensate for mutations that interfere with DSS1 binding. A. Mutation of a DSS1 interacting residue in midi-BRCA2 abrogates HR. For midi-BRCA2-2, K2630A and K2630D mutations in comparison with no BRCA2 peptide are not significantly different (*P* = 0.08 and 0.52, respectively), while compared with the respective wild-type proteins, they are (mini-BRCA2, *P*≤0.001, as in Figure 2H; midi-BRCA2-2, *P*<0.0001). B. Midi-BRCA2s with mutation of the Cter RAD51 binding site retain partial HR activity. *P*≤0.0001 compared with no BRCA2 peptide or with the respective wild-type proteins. Western blot analysis shows expression of the transiently expressed peptides.

We also investigated the importance of DSS1 binding for midi-BRCA2 function. Although the interaction between BRCA2 and DSS1 occurs within the DBD, it likely does not affect DNA binding as it occurs at a distance from the DNA binding residues (Figure 2C). The DSS1-binding mutation K2630D completely abrogates HR activity of midi-BRCA2-2 (Figure 6A), implicating DSS1 binding as an important activity of the BRCA2 peptides whether or not the peptides can bind PALB2. The K2630A mutation (Figure 2G), which does not completely abolish DSS1 binding, also substantially reduces HR activity of midi-BRCA2-2 (Figure 6A). Thus, loss of DSS1 binding and loss of DNA binding are not equivalent.

Finally, we investigated the role of the Cter RAD51-binding site in midi-BRCA2 function. Mutations at S3291 in midi-BRCA2 only partially reduce HR (≤50%, Figure 6B), similar to the two DNA binding mutations, and in contrast to the DSS1-binding mutation. Thus, the midi-BRCA2s are not strictly dependent on three of the four activities we identified to be important for mini-BRCA2 function, indicating that the ability to bind PALB2 can override the requirement for other activities. However, the requirement for DSS1 binding is maintained in the midi-BRCA2s.

### HR in the absence of the BRCA2 DBD

The substantial HR activity remaining for midi-BRCA2s mutated in the DBD or Cter domain led us to ask whether proteins deleted for these domains could be functional in HR. Gene disruption alleles with the potential to express peptides truncated after BRC3 or BRC7 permit survival of mice [37], [38], whereas those truncated before the BRC repeats do not [39], suggesting that N-terminal BRCA2 peptides retaining BRC repeats retain partial function. Further, a PARP-inhibitor insensitive BRCA2 revertant deleted for BRC6 through the DBD (PIR2) has been identified which is active in HR [40].

**Figure 7:**
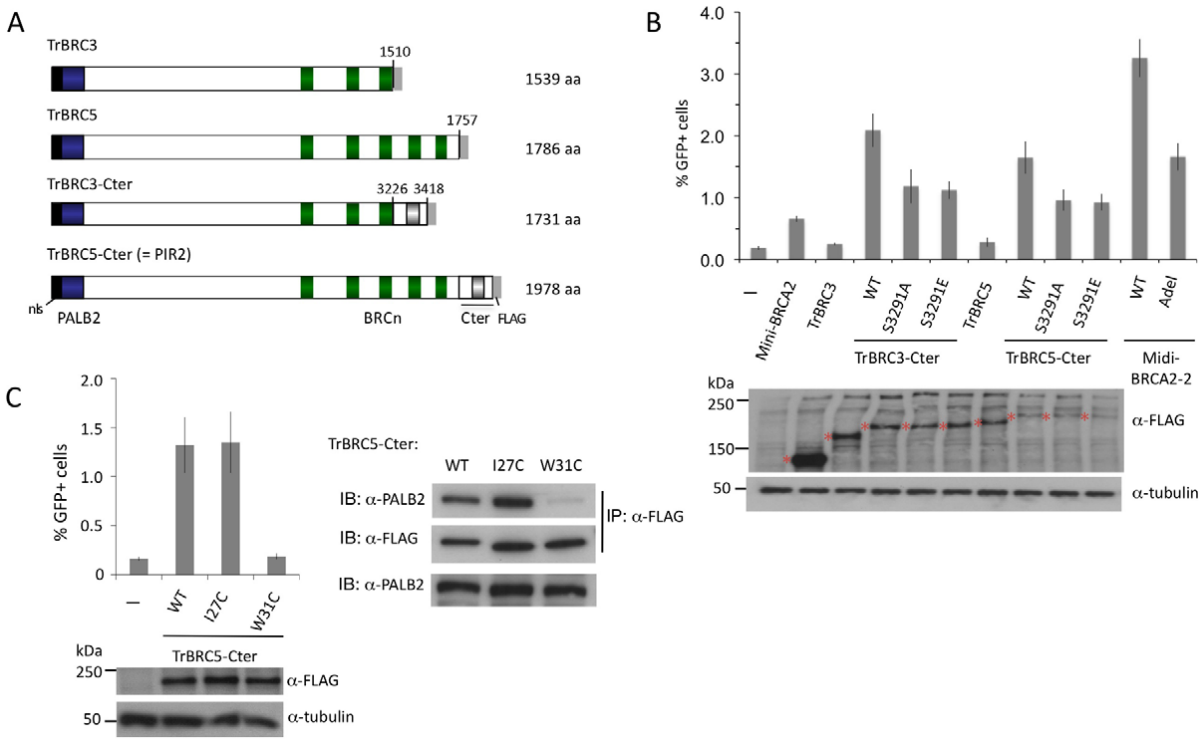
BRCA2 DBD is not required for HR in peptides that bind PALB2. A. Tr-BRCA2 domain structures. All Tr-BRCA2 peptides are deleted for C-terminal BRC repeats and the BRCA2 DBD at the indicated residues. TrBRC3 and TrBRC5 are additionally deleted for the Cter. TrBRC5-Cter corresponds to a BRCA2 peptide expressed in a PARP inhibitor-resistant revertant of Capan-1 cells (PIR2; [40]). B. TrBRC-Cter peptides are functional for HR whereas TrBRC peptides are not. The RAD51 binding site at S3291 contributes to HR activity but is not the sole determinant. *P*≤0.0001 for TrBRC-Cter compared with the respective no Cter or S3291-mutated Cter. C. Disruption of PALB2-binding by W31C mutation (right) is associated with impaired TrBRC5-Cter HR activity (left). *P* = ≤0.0001 for TrBRC5-Cter compared with the W31C mutation.

To address this, we created a series of truncated and internally deleted BRCA2 peptides (Figure 7A). Peptides truncated after BRC3 and BRC5 (i.e., TrBRC3 and TrBRC5, respectively) do not increase HR activity (Figure 7B), indicating that the PALB2 interacting site and BRC repeats are not sufficient to restore HR activity to V-C8 cells. By contrast, the addition of the Cter, creating TrBRC3-Cter, leads to substantial HR activity, demonstrating the importance of the Cter domain for HR activity of the truncations. Similarly, TrBRC5-Cter, corresponding to the previously described PIR2 revertant [40], is also active in HR. These results demonstrate that the DBD is not essential for HR and that as few as three BRC repeats can suffice for HR activity in this context. Both TrBRC3-Cter and TrBRC5-Cter show intermediate activity between that of mini-BRCA2 and midi-BRCA2-2, but similar activity to the apex deletion in midi-BRCA2-2, consistent with the DBD contributing to, but not being required for, HR.

The importance of the Cter for the function of the BRCA2 truncations led us to test the effect of mutations at the RAD51 interaction site. Both S3291A and S3291E reduce HR activity for the TrBRC-Cter peptides, but do not eliminate it (Figure 7B). These results suggest that the Cter has additional functions to RAD51 binding which enhance the activity of the truncations.

Given the absence of a DBD in the BRCA2 truncations, we speculated that PALB2 binding would be essential for their activity. To test this, we introduced the W31C mutation into TrBRC5-Cter, which substantially reduces its binding to PALB2 (Figure 7C). This mutant protein demonstrates no HR activity (Figure 7C), indicating that in the absence of DNA binding, HR activity is entirely dependent on the ability of this peptide to interact with PALB2.

### Micro-BRCA2: a minimal BRCA2

**Figure 8:**
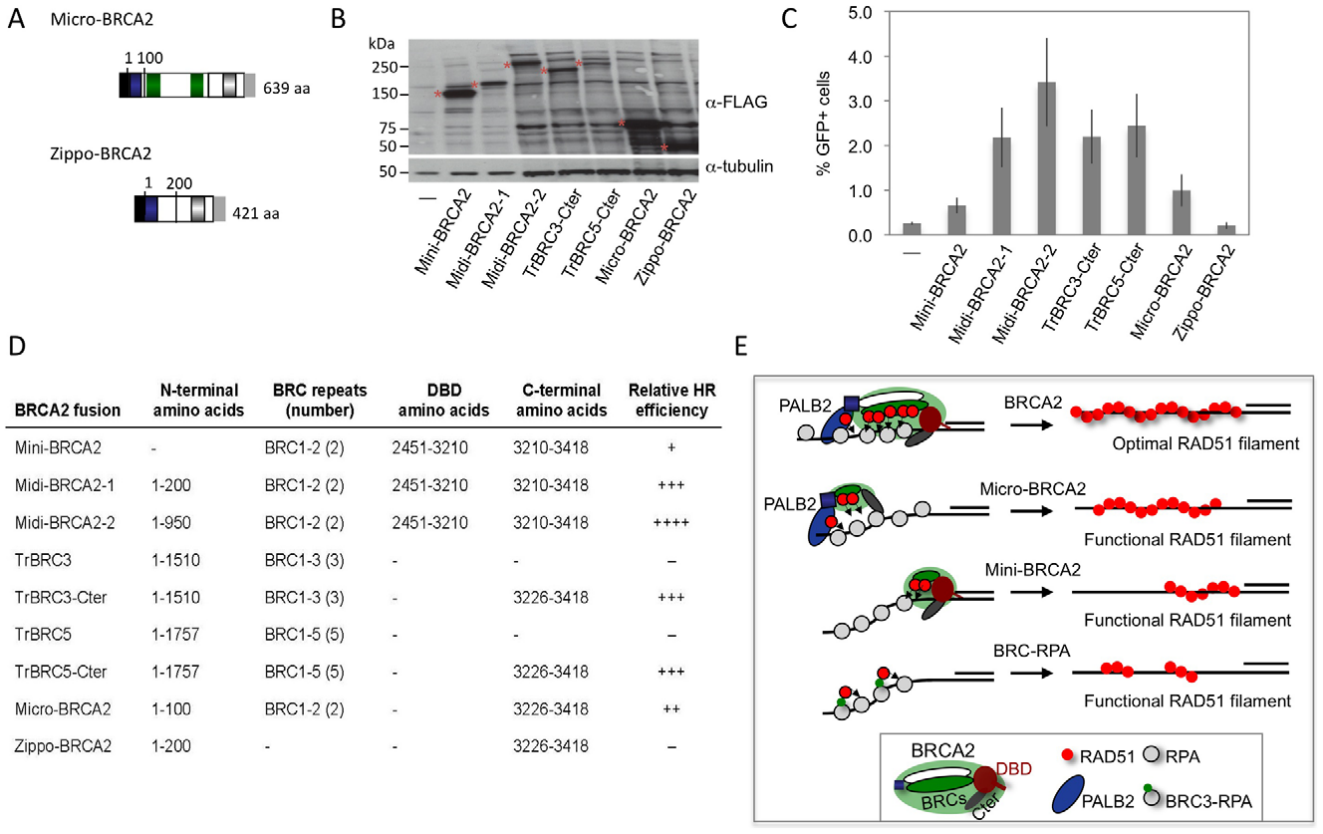
BRCA2 peptides functional in HR. A. Micro-BRCA2 domain structure. Micro-BRCA2 contains three domains – the PALB2 interaction site, BRC1–2, and the Cter, whereas zippo-BRCA2 is deleted for the BRC repeats. B. Western blot analysis showing expression of the indicated BRCA2 peptides. C. Comparison of HR activity of various BRCA2 peptides. Micro-BRCA2 is more active than mini-BRCA2 (*P* = 0.0024), whereas zippo-BRCA2 is not active. D. Summary of BRCA2 peptides and their relative HR activity. E. Schematic of peptides active in HR. While full-length BRCA2 has optimal HR activity, BRCA2 peptides can be derived which have substantial activity. Those that bind PALB2, such as micro-BRCA2, are active even with a deleted DNA binding domain (DBD). In the absence of PALB2 binding, an intact DBD is critical for HR activity. BRC fusion to RPA, however, can bypass the requirement for the DBD.

Our results with the truncated peptides suggest functional redundancy within BRCA2 and raise the possibility that BRCA2 could be pared down further yet retain HR activity. To test this, we fused the PALB2-interaction domain to two BRC repeats and the Cter, creating a micro-BRCA2 which is <20% the size of full-length BRCA2 (Figure 8A). The micro-BRCA2 is expressed (Figure 8B) and is active in HR (Figure 8C); indeed, correction of HR by the micro-BRCA2 was higher than by the mini-BRCA2 (Figure 8C). Given that the PALB2 interaction domain and the Cter are required for activity of the truncations (Figure 7B, 7C), we asked whether these two domains are sufficient to promote HR by creating another derivative peptide without BRC repeats, zippo-BRCA2 (Figure 8A). Zippo-BRCA2 is expressed (Figure 8B) but is completely inactive (Figure 8C). Thus, as with mini-BRCA2, the Cter RAD51 interaction site is not sufficient to promote HR, such that micro-BRCA2 likely has a minimal domain set required for an active BRCA2 peptide.

## Discussion

Using domain fusions, we investigated here which elements of BRCA2 are critical for HR *in vivo*. From our results, we can conclude that significant portions of BRCA2 are not essential for HR activity and, further, that functional redundancy exists within this large protein. Two types of active BRCA2 peptides can be distinguished in V-C8 cells based on the absence or presence of the BRCA2 DBD – micro-BRCA2 and mini-BRCA2, respectively (Figure 8D). In the absence of the DBD, the ability to interact with PALB2 becomes essential for detectable HR activity. Common to both forms is the requirement to bind RAD51 through the BRC repeats as well as the contribution of the Cter, which exhibits RAD51-dependent as well as independent activities.

### DNA binding in the DBD is required only for mini-BRCA2 activity

The correction of the HR defect of BRCA2-deficient cells with BRC-RPA fusions implied that the major activity of BRCA2 in HR is to deliver RAD51 to ssDNA [12] (Figure 8E). Biochemical studies support this interpretation, as full-length BRCA2 promotes RAD51 filament formation specifically on ssDNA [6]–[8]. We established that ssDNA binding is indeed required for the activity of BRCA2 peptides that lack the PALB2 interaction site: mini-BRCA2s mutated at ssDNA-binding residues in OB2 of the DBD show little HR activity. By contrast, midi-BRCA2s mutated for ssDNA binding retain substantial HR activity, as do micro/Tr-BRCA2s deleted for the DBD. Thus, ssDNA binding in the DBD becomes essential for activity in the absence of PALB2 binding. Binding to PALB2 may allow BRCA2 to interact indirectly with DNA, bypassing the need for the DBD itself (see below).

In addition to ssDNA, BRCA2 has been postulated to bind dsDNA through the tower domain 3HB, given the structural similarity of the 3HB to dsDNA binding domains in other proteins and biochemical studies with a DBD deleted for the tower [20]. Through deletion analysis, we provide evidence that the 3HB of the DBD is required for mini-BRCA2 function. Dual binding to ssDNA and dsDNA is proposed to support the recruitment of BRCA2 to ssDNA-dsDNA junctions (Figure 8E), HR intermediates arising from DNA end resection, to nucleate RAD51 filaments [6], [20], as has been shown for the BRCA2 ortholog from *Ustilago*, Brh2 [34]. Binding to ssDNA-dsDNA junctions would appear to differentiate BRCA2 from the BRC-RPA fusion, which does not have a dsDNA binding domain; the high affinity of RPA to ssDNA may allow the BRC-RPA fusion to bypass junctional binding. Unlike BRCA2 and Brh2, the BRCA2 ortholog from *C. elegans*, BRC-2, does not have a tower domain, and the single OB fold in BRC-2 is more related to OB folds in RPA than in BRCA2 [41]. Thus, it seems possible that BRC-2 may act more akin to BRC-RPA fusions, delivering RAD51 directly to ssDNA.

### DSS1 binding is required in BRCA2 peptides containing a DBD

DSS1 is known to promote HR [21], [42], even in budding yeast which does not have a BRCA2 homolog [22], raising the question as to whether the interaction with DSS1 is important for BRCA2 function. We found that interaction with DSS1 is required for HR activity of both mini and midi-BRCA2s, but not for micro/Tr-BRCA2s in which the DBD – and hence DSS1 contact residues – were deleted. Notably, the only mutation in midi-BRCA2 found to render it completely inactive for HR was one that abrogates DSS1 binding (K2630D). Thus, if the DBD is present, interaction with DSS1 is critical for BRCA2 function; however, if the DBD is absent, DSS1 binding is not required for activity, similar to what has been observed with *Ustilago* Brh2 [43].

### PALB2 interaction overrides the requirement for the DBD

An intact PALB2 interaction site in midi-BRCA2s results in substantially higher HR activity than that found with mini-BRCA2s, and partially overrides mutations that inactivate the mini-BRCA2s (i.e., in OB2, the 3HB, or the Cter). PALB2 was initially identified as an interacting protein with BRCA2 [23], but it has also been found to interact with other HR proteins, including BRCA1, with which it may form a bridge to BRCA2 [44], [45], and the RAD51 interacting protein, RAD51AP1 [46]. More recently, PALB2 has also been shown to bind DNA and RAD51 and stimulate D-loop formation *in vitro*; although this activity can function independently of BRCA2 [46], it can also synergize with a BRCA2 peptide fusion that is similar to our midi-BRCA2s [47].

That the interaction with PALB2 has such a great impact on HR suggests that BRCA2 typically functions in a complex with PALB2 and provides insight into how BRCA2 peptides lacking most of the protein, in particular micro-BRCA2, remain proficient for DSB repair (Figure 8E). Possibly, three different modes of RAD51 binding within the BRCA2/PALB2 complex participate in HR, two from BRCA2 and one from PALB2. Our results also show, however, that the absence of BRC repeats (zippo-BRCA2) cannot be compensated for by interaction with PALB2, implying that activities carried by PALB2, notably binding to RAD51, are not sufficient to restore BRCA2 peptide function.

### RAD51-dependent and -independent activities within the BRCA2 Cter

The first link between BRCA2 and RAD51 came about through the identification of an interaction between the BRCA2 Cter and RAD51 [14], with subsequent studies localizing RAD51 binding to a peptide in the middle of the Cter, specifically involving residue S3291 [17]. Importantly, this Cter peptide binds to and stabilizes RAD51 filaments, but does not bind RAD51 monomers [15]–[17]. The importance of RAD51 binding in the Cter is clear, as S3291 mutation either has a severe effect on HR activity (mini-BRCA2, midi-BRCA-2 W31C) or reduces it by ∼40% (midi-BRCA2s, TrBRC-Cter). We recently found that the S3291A mutation introduced into full-length BRCA2 by BAC mutagenesis restores HR in V-C8 cells to the same extent as wild-type BRCA2 [18], similar to results obtained in human and chicken cells [19], [48]. Thus, Cter S3291A mutation leads to a gradient of HR defects, from severe to undetectable, depending on the context.

The inability of zippo-BRCA2, which does not contain BRC repeats, to restore HR in V-C8 cells implies that the Cter is not able to deliver RAD51 to DNA. However, once RAD51 filament assembly is initiated by the BRC repeats, the Cter may stabilize filaments that otherwise would not mature sufficiently in partially HR compromised fusion-peptide backgrounds, providing an explanation for the gradient of HR activity.

Unlike the S3291A point mutation, deletion of the Cter in otherwise full-length BRCA2 leads to a moderate HR defect [4]. S3291 mutation and Cter deletion also have differential effects on HR in BRCA2 peptides (e.g., compare TrBRC with TrBRC-Cter S3291A; Figure 7B). More severe HR phenotypes with deletion of the Cter suggest that the Cter has a function in HR beyond simply binding RAD51. The only other function attributed thus far to the Cter is to promote nuclear localization of the protein [49], but given that all peptides contain an nuclear localization signal at the N terminus, the function of the Cter is unlikely to simply be to direct the protein to the nucleus. It is notable that although *Ustilago* Brh2 contains a DBD related to the BRCA2 DBD, a second DNA binding domain is present in Brh2 which is the primary determinant for DNA binding for this protein [50]. However, as yet there is no evidence that BRCA2, in particular the Cter, harbors an additional DNA binding domain.

### Functional BRCA2 peptides with reduced BRC repeat number

While BRC repeats promote the nucleation of RAD51 filaments on ssDNA [51], one and two BRC repeats are sufficient to promote HR activity of the BRC-RPA and BRCA2 peptide fusions, respectively, and adding more BRC repeats does not substantially increase HR activity. Secondary mutations in BRCA2 that reverse cisplatin and PARP inhibitor sensitivity of BRCA2 mutant cells have been identified which delete up to six BRC repeats, yet retain HR proficiency [40], [52], consistent with a lack of requirement for all eight repeats for BRCA2 function.

The BRCA2 peptide analysis presented here suggests a plausible explanation for why BRCA2 evolved to contain multiple BRC repeats: The compelling difference between midi-BRCA2-2 and full-length BRCA2 is the number of BRC repeats (two and eight BRC repeats, respectively), but only midi-BRCA2-2 shows an HR defect with Cter mutation. A larger number of BRC repeats in the full-length protein could increase the frequency of nucleation of RAD51 nucleoprotein filaments [51], allowing it to circumvent the requirement for filament stabilization by the Cter.

### Plasticity of BRCA2 structure: implications for tumorigenesis

Taken together, our results point to the plasticity of BRCA2 for HR function *in vivo*. While full-length BRCA2 is built to ensure efficient RAD51 filament formation and stabilization for optimal HR, multiple domain deletions and point mutations which compromise interactions only partially reduce HR, such that in the extreme, a peptide <20% the size of BRCA2 (micro-BRCA2) provides substantial HR activity.

This remarkable intragenic redundancy, however, may have a downside. Reduced HR efficiencies may not elicit cell death, yet may also not be sufficient to maintain genomic stability, especially when extensive repair is required. This could have profound implications for tumorigenesis. Indeed, mutations are found throughout the *BRCA2* gene in breast cancer patients (BIC database) suggesting an involvement of all functional domains in maintaining genomic stability. A large number of BRCA2 variants have been identified in the population, the significance of which to BRCA2 function and tumorigenesis are unknown. By exploiting BRCA2's functional plasticity, our system with partially compromised fusion peptides provides a fast and sensitive means to detect mutations affecting HR, many of which may be phenotypically overlooked in assays utilizing full-length BRCA2.

## Materials and Methods

### HR assays

The BRCA2 mutant V-C8 hamster cell line [28] contains a single integrated copy of DR-GFP reporter [12]. For HR assays, 5×10^6^ exponentially growing cells were collected and resuspended in 650 µl Opti-MEM media (Invitrogen) in a 0.4-cm cuvette (Biorad) with 50 µg each of the I-SceI expression vector pCBASce (or the empty pCAGGS vector) and the expression vector for a BRCA2 peptide (see below). Cells were transfected by electroporation (280 V and 1000 µF) with a Biorad Gene Pulser II. HR was measured by counting the number of GFP-positive cells using a Becton Dickinson FACSscan 48 h after electroporation. Most HR results were derived from 5 to 9 transfections from at least 3 independent experiments, except results in Figure 3C and Figure 6A which were derived from 3 transfections. *P* values were calculated using an unpaired *t* test through Graphpad software (http://www.graphpad.com/quickcalcs/ttest1.cfm).

### Plasmids

Vectors expressing BRCA2 peptides have a pCAGGS backbone. All peptides contain an N-terminal nuclear localization signal and a C-terminal FLAG epitope tag [12]. Mini-BRCA2 vectors were derived by modifying vectors encoding BRC-RPA fusions [12], such that individual BRC motifs correspond to amino acids 996–1064 (BRC1), 1206–1274 (BRC2), 1415–1483 (BRC3), and 1511–1575 (BRC4) of human BRCA2 and multiple BRC motifs correspond to amino acids 923–1252 (BRC1–2) and 923–1563 (BRC1–4) of mouse BRCA2. To generate the mini-BRCA2s, the vector fragment encoding RPA70 in the BRC-RPA fusion was swapped with a fragment encoding the BRCA2 DBD (residues 2451–3210) or the DBD through the Cter (residues 2451–3418). BRCA2 fragments were amplified by PCR using a human BRCA2 full-length cDNA as a template and a C-terminal primer designed to maintain the FLAG epitope.

Midi-BRCA2 vectors were generated by introducing a fragment encoding human BRCA2 residues 1 to 200 (midi-BRCA2-1) or 1 to 950 (midi-BRCA2-2) at a site located between the nuclear localization signal sequence and BRC1–2 in mini-BRCA2. TrBRC peptides encode human BRCA2 until residue 1510 (TrBRC3) or 1757 (TrBRC5), while the TrBRC-Cter peptides additionally encode residues 3226–3418 of human BRCA2.

Adel and Tdel were generated by PCR to delete BRCA2 residues 2889–2959 (Tdel) and 2889–2919 (Adel) and insert short peptide linkers SGSGSGS (Tdel) and SGS (Adel). Tdel is based on the BRCA2 peptide, ΔTower, used for biochemical studies in which much of the tower was cleaved from the mouse BRCA2 DBD using TEV protease [20]. In ΔTower, tower alpha helix 1 (Ta1) is maintained but the other tower helices (Ta2–Ta5) are removed [20]. In Tdel, the approximately 20 amino acids after Ta1 that are disordered in the structure are maintained and followed by the 7 amino acid peptide linker, which then connects with the base of the tower prior to OB2 at the same position of TEV protease cleavage. In Adel, only the 3HB helices (Ta2–Ta4) are deleted.

The DSS1 expression vector was generated by inserting the human DSS1 cDNA into pCAGGS with an N-terminal c-myc tag. The PALB2 expression vector was generated by inserting human PALB2 into a modified pEGFP-N3 (Clontech) vector with an added N-terminal c-myc tag. The resulting construct expresses PALB2 with a myc tag at the N terminus and a GFP tag fused at the C terminus.

### Protein analysis

BRCA2 peptide expression was examined 48 h after electroporation by Western blotting using 40–100 µg of total lysate separated by 10% Tris-Glycine or 4–12% Bis-Tris polyacrylamide gel electrophoresis (Invitrogen). Membranes were probed with anti-FLAG-M2 antibody (A8592, Sigma) or anti-alpha-tubulin antibody (T6074, Sigma) to assess protein loading. For co-immunoprecipitations, V-C8 cells were electroporated as above with 50 µg each of the expression vectors for the BRCA2 peptide and PALB2 or DSS1. Cells were collected ∼40 h after electroporation and resuspended in NETN buffer (150 mM NaCl, 1 mM EDTA, 50 mM Tris-HCl, pH 8.0, 0.5% NP40) containing protease inhibitor (Complete Mini Roche). Cell lysates were obtained by repeated cycles of freezing (dry ice/ethanol) and thawing (warm water). For PALB2, protein extracts from cells expressing midi-BRCA2-1 and TrBRC5-Cter (600 µg) and midi-BRCA2-2 (450 µg) were incubated with 40 µl EZview Red ANTI-FLAG M2 Affinity Gel (Sigma F2426) for 5–6 h at 4°C. Proteins were eluted with SDS-PAGE sample buffer and loaded on a BIORAD ready gel 7.5% Tris-HCl (100–150 V, ∼2 h) and then transferred to a nitrocellulose membrane overnight at 4°C. Membranes were blocked using TBS-Tween 20–5% BioRad Non-Fat Dry Milk and probed with anti-PALB2 antibody M11 raised against amino acids 601 to 880 of human PALB2 and monoclonal anti-FLAG M2-Peroxidase (HRP) antibodies. For Western blotting, 50 µg of protein were loaded on a BIORAD ready gel 7.5% Tris-HCl. For DSS1, co-immunoprecipitations were performed with 100–500 µg whole cell extract and 20 µl of anti–c-myc Agarose Affinity Gel (A7470, Sigma) for 4 h to overnight at 4°C. Immune complexes were washed with lysis buffer and separated by 4–12% Bis-Tris polyacrylamide gel electrophoresis. Membranes were probed with anti-FLAG-M2 antibody.

## References

[1] San Filippo J, Sung P, Klein H (2008). Mechanism of eukaryotic homologous recombination.. Annu Rev Biochem.

[2] Moynahan ME, Jasin M (2010). Mitotic homologous recombination maintains genomic stability and suppresses tumorigenesis.. Nat Rev Mol Cell Biol.

[3] Mimitou EP, Symington LS (2009). Nucleases and helicases take center stage in homologous recombination.. Trends Biochem Sci.

[4] Moynahan ME, Pierce AJ, Jasin M (2001). BRCA2 Is required for homology-directed repair of chromosomal breaks.. Mol Cell.

[5] Chen CF, Chen PL, Zhong Q, Sharp ZD, Lee WH (1999). Expression of BRC repeats in breast cancer cells disrupts the BRCA2-Rad51 complex and leads to radiation hypersensitivity and loss of G(2)/M checkpoint control.. J Biol Chem.

[6] Jensen RB, Carreira A, Kowalczykowski SC (2010). Purified human BRCA2 stimulates RAD51-mediated recombination.. Nature.

[7] Liu J, Doty T, Gibson B, Heyer WD (2010). Human BRCA2 protein promotes RAD51 filament formation on RPA-covered single-stranded DNA.. Nat Struct Mol Biol.

[8] Thorslund T, McIlwraith MJ, Compton SA, Lekomtsev S, Petronczki M (2010). The breast cancer tumor suppressor BRCA2 promotes the specific targeting of RAD51 to single-stranded DNA.. Nat Struct Mol Biol.

[9] Davies AA, Masson JY, McIlwraith MJ, Stasiak AZ, Stasiak A (2001). Role of BRCA2 in control of the RAD51 recombination and DNA repair protein.. Mol Cell.

[10] Chen Z, Yang H, Pavletich NP (2008). Mechanism of homologous recombination from the RecA-ssDNA/dsDNA structures.. Nature.

[11] Stark JM, Hu P, Pierce AJ, Moynahan ME, Ellis N (2002). ATP hydrolysis by mammalian RAD51 has a key role during homology- directed DNA repair.. J Biol Chem.

[12] Saeki H, Siaud N, Christ N, Wiegant WW, van Buul PP (2006). Suppression of the DNA repair defects of BRCA2-deficient cells with heterologous protein fusions.. Proc Natl Acad Sci U S A.

[13] Pellegrini L, Yu DS, Lo T, Anand S, Lee M (2002). Insights into DNA recombination from the structure of a RAD51-BRCA2 complex.. Nature.

[14] Sharan SK, Morimatsu M, Albrecht U, Lim DS, Regel E (1997). Embryonic lethality and radiation hypersensitivity mediated by Rad51 in mice lacking Brca2.. Nature.

[15] Davies OR, Pellegrini L (2007). Interaction with the BRCA2 C terminus protects RAD51-DNA filaments from disassembly by BRC repeats.. Nat Struct Mol Biol.

[16] Esashi F, Galkin VE, Yu X, Egelman EH, West SC (2007). Stabilization of RAD51 nucleoprotein filaments by the C-terminal region of BRCA2.. Nat Struct Mol Biol.

[17] Esashi F, Christ N, Gannon J, Liu Y, Hunt T (2005). CDK-dependent phosphorylation of BRCA2 as a regulatory mechanism for recombinational repair.. Nature.

[18] Schlacher K, Christ N, Siaud N, Egashira A, Wu H (2011). Double-Strand Break Repair-Independent Role for BRCA2 in Blocking Stalled Replication Fork Degradation by MRE11.. Cell.

[19] Ayoub N, Rajendra E, Su X, Jeyasekharan AD, Mahen R (2009). The carboxyl terminus of Brca2 links the disassembly of Rad51 complexes to mitotic entry.. Curr Biol.

[20] Yang H, Jeffrey PD, Miller J, Kinnucan E, Sun Y (2002). BRCA2 Function in DNA Binding and Recombination from a BRCA2-DSS1-ssDNA Structure.. Science.

[21] Gudmundsdottir K, Lord CJ, Witt E, Tutt AN, Ashworth A (2004). DSS1 is required for RAD51 focus formation and genomic stability in mammalian cells.. EMBO Rep.

[22] Krogan NJ, Lam MH, Fillingham J, Keogh MC, Gebbia M (2004). Proteasome involvement in the repair of DNA double-strand breaks.. Mol Cell.

[23] Xia B, Sheng Q, Nakanishi K, Ohashi A, Wu J (2006). Control of BRCA2 cellular and clinical functions by a nuclear partner, PALB2.. Mol Cell.

[24] Tischkowitz M, Xia B (2010). PALB2/FANCN: recombining cancer and Fanconi anemia.. Cancer Res.

[25] Casadei S, Norquist BM, Walsh T, Stray S, Mandell JB (2011). Contribution of inherited mutations in the BRCA2-interacting protein PALB2 to familial breast cancer.. Cancer Res.

[26] Auerbach AD (2009). Fanconi anemia and its diagnosis.. Mutat Res.

[27] Christ N, Moynahan ME, Jasin M, Rothstein R (2007). BRCA2: safeguarding the genome through homologous recombination.. Molecular Genetics of Recombination.

[28] Kraakman-van der Zwet M, Overkamp WJ, van Lange RE, Essers J, van Duijn-Goedhart A (2002). Brca2 (XRCC11) deficiency results in radioresistant DNA synthesis and a higher frequency of spontaneous deletions.. Mol Cell Biol.

[29] Pierce AJ, Johnson RD, Thompson LH, Jasin M (1999). XRCC3 promotes homology-directed repair of DNA damage in mammalian cells.. Genes and Development.

[30] San Filippo J, Chi P, Sehorn MG, Etchin J, Krejci L (2006). Recombination mediator and Rad51 targeting activities of a human BRCA2 polypeptide.. J Biol Chem.

[31] Wong AKC, Pero R, Ormonde PA, Tavtigian SV, Bartel PL (1997). RAD51 interacts with the evolutionarily conserved BRC motifs in the human breast cancer susceptibility gene brca2.. J Biol Chem.

[32] Galkin VE, Esashi F, Yu X, Yang S, West SC (2005). BRCA2 BRC motifs bind RAD51-DNA filaments.. Proc Natl Acad Sci U S A.

[33] Bochkarev A, Bochkareva E (2004). From RPA to BRCA2: lessons from single-stranded DNA binding by the OB-fold.. Curr Opin Struct Biol.

[34] Yang H, Li Q, Fan J, Holloman WK, Pavletich NP (2005). The BRCA2 homologue Brh2 nucleates RAD51 filament formation at a dsDNA-ssDNA junction.. Nature.

[35] Marston NJ, Richards WJ, Hughes D, Bertwistle D, Marshall CJ (1999). Interaction between the product of the breast cancer susceptibility gene BRCA2 and DSS1, a protein functionally conserved from yeast to mammals.. Mol Cell Biol.

[36] Oliver AW, Swift S, Lord CJ, Ashworth A, Pearl LH (2009). Structural basis for recruitment of BRCA2 by PALB2.. EMBO Rep.

[37] Friedman LS, Thistlethwaite FC, Patel KJ, Yu VP, Lee H (1998). Thymic lymphomas in mice with a truncating mutation in Brca2.. Cancer Res.

[38] Connor F, Bertwistle D, Mee PJ, Ross GM, Swift S (1997). Tumorigenesis and a DNA repair defect in mice with a truncating Brca2 mutation.. Nat Genet.

[39] Moynahan ME (2002). The cancer connection: BRCA1 and BRCA2 tumor suppression in mice and humans.. Oncogene.

[40] Edwards SL, Brough R, Lord CJ, Natrajan R, Vatcheva R (2008). Resistance to therapy caused by intragenic deletion in BRCA2.. Nature.

[41] Martin JS, Winkelmann N, Petalcorin MI, McIlwraith MJ, Boulton SJ (2005). RAD-51-dependent and -independent roles of a Caenorhabditis elegans BRCA2-related protein during DNA double-strand break repair.. Mol Cell Biol.

[42] Kristensen CN, Bystol KM, Li B, Serrano L, Brenneman MA (2010). Depletion of DSS1 protein disables homologous recombinational repair in human cells.. Mutat Res.

[43] Kojic M, Zhou Q, Lisby M, Holloman WK (2005). Brh2-Dss1 Interplay Enables Properly Controlled Recombination in Ustilago maydis.. Mol Cell Biol.

[44] Sy SM, Huen MS, Chen J (2009). PALB2 is an integral component of the BRCA complex required for homologous recombination repair.. Proc Natl Acad Sci U S A.

[45] Zhang F, Ma J, Wu J, Ye L, Cai H (2009). PALB2 links BRCA1 and BRCA2 in the DNA-damage response.. Curr Biol.

[46] Dray E, Etchin J, Wiese C, Saro D, Williams GJ (2010). Enhancement of RAD51 recombinase activity by the tumor suppressor PALB2.. Nat Struct Mol Biol.

[47] Buisson R, Dion-Cote AM, Coulombe Y, Launay H, Cai H (2010). Cooperation of breast cancer proteins PALB2 and piccolo BRCA2 in stimulating homologous recombination.. Nat Struct Mol Biol.

[48] Hucl T, Rago C, Gallmeier E, Brody JR, Gorospe M (2008). A syngeneic variance library for functional annotation of human variation: application to BRCA2.. Cancer Res.

[49] Spain BH, Larson CJ, Shihabuddin LS, Gage FH, Verma IM (1999). Truncated BRCA2 is cytoplasmic: implications for cancer-linked mutations.. Proc Natl Acad Sci USA.

[50] Zhou Q, Kojic M, Holloman WK (2009). DNA-binding Domain within the Brh2 N Terminus Is the Primary Interaction Site for Association with DNA.. J Biol Chem.

[51] Carreira A, Hilario J, Amitani I, Baskin RJ, Shivji MK (2009). The BRC repeats of BRCA2 modulate the DNA-binding selectivity of RAD51.. Cell.

[52] Sakai W, Swisher EM, Karlan BY, Agarwal MK, Higgins J (2008). Secondary mutations as a mechanism of cisplatin resistance in BRCA2-mutated cancers.. Nature.

[53] Sharan SK, Pyle A, Coppola V, Babus J, Swaminathan S (2004). BRCA2 deficiency in mice leads to meiotic impairment and infertility.. Development.

